# Occupational related heat stress exposure and heat-related symptoms among sugarcane workers in Thailand

**DOI:** 10.1007/s00420-026-02216-4

**Published:** 2026-06-16

**Authors:** Martie van Tongeren, Tadpong Tantipanjaporn, Andrew Povey, Holly A. Shiels, Matthew Gittins

**Affiliations:** 1 Division of Occupational Health and Safety, Faculty of Public Health, Naresuan University, 99 Moo 9, Thapo Sub-District, Muang District, Phitsanulok City, 65000 Thailand; 2 Centre for Occupational and Environmental Health, School of Health Sciences, Faculty of Biology, Medicine and Health, University of Manchester, Ellen Wilkinson Building (Block C), Oxford Road, Manchester, M13 9PL UK; 3 Division of Cardiovascular Sciences, School of Medical Sciences, Faculty of Biology, Medicine and Health, Core Technology Facility, University of Manchester, 46 Grafton Street, Manchester, M13 9NT UK; 4 Centre for Biostatistics, School of Health Sciences, Faculty of Biology, Medicine and Health, University of Manchester, Jean McFarlane Building, Oxford Road, Manchester, M13 9PL UK; 5 Thomas Ashton Institute for Risk and Regulatory Research, University of Manchester, Nancy Rothwell Building, Oxford Road, Manchester, M13 9PL UK

**Keywords:** Heat stress exposure, Wet bulb globe temperature, Heat-related illness, Agricultural workers, Harvesting period, Global warming

## Abstract

**Objective:**

Thai sugarcane workers often work in very hot, humid conditions. This study investigated the effect of heat stress exposure on heat-related symptoms and severe heat-related symptoms associated with heat stroke in Thai sugarcane workers.

**Methods:**

Sugarcane workers in Nakhon Sawan Province, Thailand, recruited during cooler and hotter months of 2023 provided information on demographics, health behaviours, working conditions, and 21 heat-related symptoms, including 8 severe heat-related symptoms. Environmental heat was assessed using Wet-Bulb Globe Temperature (WBGT). Full shift time-weighted average effective WBGTs (WBGT_eff_-FS-TWA) were estimated and adjusted for clothing to represent heat stress exposure. Associations between heat stress exposure and symptom counts were examined using generalised negative binomial regression, adjusting for available confounders. Generalised binary logistic regression assessed associations between heat stress exposure and having ≥ 10 heat-related symptoms or ≥ 3 severe heat-related symptoms.

**Results:**

The mean measured WBGT was 28.9 ± 2.6 °C. The mean WBGT_eff_-FS-TWA was 31.3 ± 2.8 °C, with more than 40% exposed to 32.0–34.0 °C. Of the 295 participants (165 males, 130 females), there were no significant gender differences observed in the risk of heat-related symptoms. Ninety-eight percent experienced ≥ 1 heat-related symptom, and 74% reported ≥ 1 severe heat-related symptom. Relative risk (RR (95%CI)) for the number of heat-related symptoms was 1.10 (1.08, 1.13) per 1 °C increase in WBGT_eff_-FS-TWA, whereas for severe heat-related symptoms, the RR was 1.19 (1.14, 1.24). Odds ratio (OR) for ≥ 10 heat-related symptoms was 1.50 (1.29, 1.75) per 1 °C; for ≥ 3 severe heat-related symptoms OR was 1.49 (1.30, 1.72).

**Conclusion:**

Sugarcane workers in Thailand report very high prevalence of heat-related symptoms. The number of reported heat-related and severe heat-related symptoms increases significantly as temperature increases. These results highlight the urgent need to protect sugarcane and other agricultural workers from excessive heat stress.

**Supplementary Information:**

The online version contains supplementary material available at 10.1007/s00420-026-02216-4.

## Introduction

Increased global temperature induced by climate change is a major concern. The previous decade (2015–2024) was the world’s warmest, and the year 2024 was the warmest year in history since records began (National Centers for Environmental Information, 2025). Countries located in the tropics already have a climate which is hot, wet, and humid, and Thailand is considered fully tropical (World Population Review 2025). In 2024, the average annual temperature in Thailand was 28.5 °C, which was the highest average annual temperature recorded in the previous 73 years (1951—2024) (Thai Meteorological Department, 2024).

Sugarcane is a vital economic crop in Thailand (National Statistical Office (Thailand, 2024). The Ministry of Industry’s Cane and Sugar Board of Thailand reported that during the last 10 years (2014 – 2023), the annual sugarcane cultivation area in Thailand was over 16 billion square metres, spanning 47 Thai provinces. In 2023, Thailand’s Nakhon Sawan Province had the largest sugarcane cultivation area of any Thai province, at 1.3 billion square metres with approximately 6.4 million tons of harvested sugarcane sent to sugar mills (Office of the Cane and Sugar Board 2024). The sugarcane harvesting period in Thailand is normally four months, from December to March. This period covers Thailand’s cool season (mid-October to mid-February) and hot season (mid-February to mid-May) (Boonruksa et al. 2020).

Sugarcane harvesters need to work in very hot and humid conditions while performing heavy work in the fields; their heat stress exposure exceeds the standards established by Thailand and American Conference of Governmental Industrial Hygienists (ACGIH) (Boonruksa et al. 2020; Pundee et al. 2021; Wichatorn & Chaiklieng 2022a). Worsening matters, this population is an informal workforce to which Thai regulatory standards of occupational health and safety are not officially applicable (Boonruksa et al. 2020). With global temperatures rising steadily due to climate change, occupational heat stress exposure in agricultural populations is also increasing continuously. This highlights the need for comprehensive research investigating the effects of increased heat stress exposure on heat-related symptoms in this population. There are several research gaps that need to be addressed. For example, while it is well known that sugarcane workers are at high risk of heat-related illness (Kiatkitroj et al. 2022), there is a lack of region-specific evidence on how increasing heat stress exposure affects the severity of heat symptoms among workers in a hot and humid country like Thailand. Because previous studies define heat-related symptoms differently, we first conducted a literature review for all heat-related symptoms; in our current study, we include all heat-related symptoms that were consistently reported in previous studies. None of the previous studies have focused specifically on heat stroke symptoms, which are potentially life-threatening (Grubenhoff et al. 2007; Thawillarp et al. 2015). This study, however, investigates these symptoms specifically. Focussing on this specific category is a novel contribution of our study, as an extensive search of the literature indicates a lack of research on severe symptoms in this occupational group. This distinction is vital for developing effective, life-saving interventions for vulnerable workers. This study sought to address these gaps by collecting and analysing data specific to Thai sugarcane workers during the cool and hot harvesting period. The primary aim of this study was to determine the effects of heat stress, measured using the wet bulb globe temperature (WBGT) index, on overall heat-related symptoms. Additionally, the secondary aim was to investigate the effects on symptoms typically associated with heat stroke, hereafter referred to as severe heat-related symptoms. Here heat-related symptoms refer to 21 symptoms of acute heat-related illnesses while severe heat-related symptoms refer to the most severe heat-related symptoms including dizziness, headache, nausea or vomiting, confusion, dry and cracking skin, rapid pulse, unconsciousness, and very high body temperature or fever, as according to established guidelines and findings from previous studies (Centers for Disease Control and Prevention 2024; Di Lorenzo et al. 2009; Leon & Bouchama 2015; National Institute for Occupational Safety and Health 2016).

## Materials and methods

### Study design and setting

Details for the study design, setting, period, participant selection, heat stress assessment, and questionnaire have been described previously (Tantipanjaporn et al. 2025). This cross-sectional study was repeated at two timepoints, one during the cooler months (mid-January to mid-February 2023) and the one during the hotter month (March 2023) of the harvesting period. Sugarcane workers, employed in sugarcane fields in Thailand’s Nakhon Sawan Province were recruited. Sugarcane workers from 13 camps (locations where they live during the harvesting period) in Khao Kala Sub-district, Phayuha Khiri District, Nakhon Sawan Province participated in this study. The district and sub-district were chosen randomly from all districts and sub-districts in the province, while the camps in Khao Kala Sub-district were selected using convenience sampling. The camps were visited in a generally North to South direction, starting in the northernmost village of Ban Huai Bong. Inclusion criteria included being at least 18 years of age, having the ability to read, write, and communicate in Thai, and actively working on the day of the researcher’s visit. We did not exclude participants who had medical conditions; however, these were recorded and accounted for in the analysis. Because the sugarcane harvesting period spans both the cool and hot seasons, all data collection, including heat stress measurements and questionnaires was conducted over 14 non-consecutive days during the cooler months (January 18 to February 10, 2023) and 11 consecutive days during the hotter month (March 11–20, 2023), for a total of 25 days. The selected days depended on the agreement of farmers (sugarcane field owners) and participants. There was no overlap of participants between testing days, either within or between seasons.

### Heat stress measurements

The Wet Bulb Globe Temperature (WBGT) index was used to assess heat exposure among the participants, following ISO 7243:2017 (International Organization for Standardization 2017). The WBGT instruments (Brand: TSI Quest, Model: QUESTemp 34, Calibrated date: 9 January 2023) were placed in each field for a full work shift (approximately 06:00 to 17:00). The instruments were calibrated daily by plugging the verification module into the sensor at the top of the unit both before and after measurements. The instruments logged the data every 5 min throughout the work shift, and then automatically provided natural wet-bulb temperature (Tnwb), dry-bulb temperature (Tdb), globe temperature (Tg), relative humidity (Rh), and measured WBGT. Because the workers worked outdoors, the average WBGT Outdoor was calculated as WBGT (outdoor) = 0.7 Tnwb + 0.2 Tg + 0.1 Ta (American Conference of Governmental Industrial Hygienists 2025). To account for the effect of clothing, effective WBGT was applied and calculated as Effective WBGT = the measured WBGT + Clothing Adjustment Factor (CAF). The clothing worn by each worker was recorded through direct observation on the measurement day and by self-report using a 7-day recall questionnaire. According to the ACGIH guidelines, this study applied a CAF of 0 °C for a standard cotton shirt and trousers, and 3 °C for double layer cloth (woven) clothing (American Conference of Governmental Industrial Hygienists 2025). For workers wearing three layers, a CAF of 3 °C was also applied because there is no CAF guideline for more than two layers of clothes. Finally, an individual full work shift time-weighted average effective WBGT (WBGT_eff_-FS-TWA) was determined for each worker.

### Questionnaire

A questionnaire was developed based on previous studies (Boonruksa et al. 2020; Kiatkitroj et al. 2022; Spector et al. 2015). The questionnaire consisted of five parts: demographic characteristics, health behaviours, working conditions, the use of clothing, and the occurrence of 21 heat-related symptoms in the 7 days prior to the data collection day. The participants were asked if they had experienced any of the following 21 symptoms at work: rash on skin, blisters on skin, swelling of hands or feet, muscle cramps, fainting, dizziness, heavy sweating, weakness/fatigue, headache, thirst, irritability, elevated body temperature, dry mouth, urogenital issues such as oliguria or dysuria, nausea or vomiting, confusion, dry and cracking skin, rapid pulse, difficulty in breathing, unconsciousness, and very high body temperature or fever. To complete the questionnaire, participants were interviewed by a research team of Field Representatives (FRs) who were trained and supervised by the Lead Researcher (LR, T. Tantipanjaporn). The FRs were trained by the LR to understand the questionnaire in detail. Given the participants were Thai, the questionnaire underwent a thorough translation and validation process. This included forward translation into Thai, synthesis of the translated versions, backward translation into English, a comparative review of the original and back-translated versions, and an assessment of content validity using Item-Objective Congruence (IOC) by seven experts. The average IOC value of the questionnaire was 0.95, indicating good content validity.

### Variables of interest

A directed acyclic graph (DAG) was created using the DAGitty programme (version 3.1) to present our best understanding of the hypothetical causal relationships between primary explanatory variable and the outcome variable, along with known confounding variables (Figure S1 in Supplementary Information)***.*** To estimate the “total” effect of heat stress exposure measured using WBGT_eff_-FS-TWA on heat-related symptoms in the previous 7 days, minimal sufficient adjustment sets included all known identified potential confounders.

### Outcome and explanatory variables

The outcome of interest in this study was all heat-related symptoms, and this was considered in three ways. Initially, the main analysis focused on a count of the reported number of heat-related symptoms. For the secondary analyses, dichotomous heat-related symptom variables were created based on two thresholds: the 75^th^ percentile cut-off point (10 or more symptoms), examining severe cases, and the median cut-off point (6 or more symptoms), examining moderate to severe cases.

Further separate analyses were carried out for a severe heat-related symptoms (a specific group of eight symptoms characteristic of heat stroke), which included dizziness, headache, nausea or vomiting, confusion, dry and cracking skin, rapid pulse, unconsciousness, and very high body temperature or fever (Centers for Disease Control and Prevention 2024; Di Lorenzo et al. 2009; Leon & Bouchama 2015; National Institute for Occupational Safety and Health 2016). Similar to the heat-related symptoms, the analyses for severe heat-related symptoms were carried out in three ways. The primary analyses used the number of severe heat-related symptoms as the outcome variable, whilst for the secondary analyses dichotomous variables were created using the 75^th^ percentile (3 or more symptoms) and median (2 or more symptoms) as the cut-off points. The main exposure variable used was WBGT_eff_-FS-TWA, considered as a continuous variable.

### Confounding variables

This study included five main groups of confounding variables. The variables included in the analysis were selected based on the DAG constructed prior to data analysis, in order that the final explanatory effect represent hypothesised causal relationships. The socio-demographic variables are gender, age, education, and work experience. The health metrics variables are body mass index (BMI) and medical conditions. The health behaviour variables are alcohol drinking, smoking, caffeine intake, and average sleep duration. The mitigating variables are factors that can change the effects of heat stress exposure on outcomes. The following potential mitigating variables were considered: total duration of breaks, total fluid intake at work, and hours worked during a week. The only working task is the main job task in the previous 7 days, which was divided into two categories. The first category includes harvesting only or harvesting with one additional high-physical-level task, i.e., levelling sugarcane on a track. The second category involves harvesting with one additional low-physical-level task, i.e., counting sugarcane, driving a tractor, or operating a sugarcane leaf remover.

### Data analysis

IBM SPSS Statistics for Windows, version 29.0.2.0 (IBM Corp., 2023), was applied to analyse data. Descriptive statistics were used to characterise the data in terms of frequency, percentage, mean, standard deviation (S.D.), and minimum and maximum values. Chi-square tests were applied to evaluate differences in the distribution of socio-demographic and occupational characteristics across work tasks and clothing layers. To analyse a count outcome (number of symptoms), generalised linear negative binomial models reporting relative risks (RR) were used while for dichotomous outcome variables generalised binary logistic models reporting odds ratios (OR) were used. This study reported the results from unadjusted models and adjusted models including all identified confounders. To evaluate whether the association between WBGT_eff_-FS-TWA and heat-related symptoms varied by gender, an interaction term between the two variables was incorporated. Additional models exploring the effect of specific groups of confounders in a step-by-step process were also investigated, and the results are presented in Table S1 in Supplementary Information. As part of an exploratory analysis, this study additionally explored whether there is any evidence of a non-linear relationship by applying piecewise double linear relationships at three different cut-off points of 30 °C, 29 °C, and 28 °C. These cut-off points were selected using a data-driven approach based on a visual inspection of scatter plots: the first for heat-related symptoms and the second for severe heat-related symptoms (Figures S2 and S3, respectively (Supplementary Information)). Also, the WBGT_eff_-FS-TWA was transformed into categorical form to investigate whether there were potential non-linear trends.

## Results

### Study participant profile

Of the 300 participants recruited, five were excluded from the analysis due to incomplete questionnaires, resulting in a final sample of 295 (148 participants in the cooler months and 147 in the hotter month). One participant answered “prefer not to say” for gender, and four responded “do not recall” to the question about drinking. Information on participant characteristics is shown in Table 1. The majority were male (55.9%). Their average age was 41 years. The majority of the participants only finished primary school or less. One-half of the participants (52.5%) had less than five years of work experience in sugarcane harvesting. Twenty-seven percent of them had Class I obesity, according to the World Health Organisation’s Asia–Pacific BMI classification. Medical conditions were found in about 18% of the total group. Approximately half of the participants reported being current alcohol drinkers, and a similar proportion were smokers. Almost all participants reported consuming caffeine. Their average sleep duration was approximately 8 h (range: 4 – 12 h). The participants’ mean total break duration per shift was 1.3 ± 0.9 h, and their mean total liquid consumption at work was 3.4 ± 1.8 L. The participants harvested almost every day, for an average of 6.9 days a week (range: 1 – 7 days), and the average number of hours worked per week was 69.6 (range: 10 – 91 h). Their most common primary job task in the previous 7 days was “sugarcane harvesting only” (93.6%).

**Table 1:** General characteristics of the study participants (n = 295)

Descriptor	Category	Result
*Socio-demographic variables*		
Gender, n (%)	Male Female	165 (55.9) 130 (44.1)
Age (years), n (%)	< 30 30–39 40–49 ≥ 50 Mean ± S.D Range	58 (19.7) 66 (22.3) 84 (28.5) 87 (29.5) 41.3 ± 11.7 18.0 – 81.0
Educational level, n (%)	≤ Primary school (6 years) Middle school (9 years) ≥ High school or technical school (12 years)	199 (67.5) 65 (22.0) 31 (10.5)
Work experience (years), n (%)	< 5 5–10 > 10 Mean ± S.D Range	155 (52.5) 81 (27.5) 59 (20.0) 7.6 ± 7.4 4 d – 36.0 y
*Health metric variables*		
BMI, n (%)	Underweight (< 18.5 kg/m^2^) Normal weight (18.5–22.9 kg/m^2^) Overweight (23.0–24.9 kg/m^2^) Obese I (≥ 25.0 kg/m^2^) Mean ± S.D Range	35 (11.9) 141 (47.8) 39 (13.2) 80 (27.1) 22.9 ± 4.4 11.7 – 39.6
Medical conditions, n (%)	No Yes	241 (81.7) 54 (18.3)
*Health behaviour variables*		
Current alcohol drinking, n (%)	No Yes	143 (48.5) 152 (51.5)
Current smoking, n (%)	No Yes	154 (52.2) 141 (47.8)
Caffeine intake*, n (%)	No (no intake of coffee/tea/soft drink/energy drink) Yes (any intake of coffee/tea/soft drink/energy drink)	12 (4.1) 283 (95.9)
Average sleep duration (hours)	Mean ± S.D Range	8.3 ± 1.3 4.0 – 12.0
*Mitigating variables*		
Total duration of breaks per shift (hours)	Mean ± S.D Range	1.3 ± 0.9 0.1 – 4.2
Total volume of liquids consumed at work (litres)	Mean ± S.D Range	3.4 ± 1.8 0.5 – 10.3
Hours worked per day (hours)	Mean ± S.D Range	10.1 ± 1.2 7.0 – 13.0
Days worked per week (days)	Mean ± S.D Range	6.9 ± 0.6 1.0 – 7.0
Hours worked per week (hours)	Mean ± S.D Range	69.6 ± 10.4 10.0 – 91.0
*Working task*		
Main job task in the previous 7 days, n (%)	Sugarcane harvesting only Sugarcane harvesting plus one additional high-physical-level task, i.e., levelling sugarcane on a track Sugarcane harvesting plus one additional low-physical-level task, i.e., counting sugarcane, driving a tractor, or operating a sugarcane leaf remover	276 (93.6) 5 (1.7) 14 (4.7)

^*^Caffeine intake referred to whether the participants drink coffee, tea, soft drinks (treated as mostly caffeinated), or energy drinks

### Sugarcane harvesting

There are two main methods for harvesting sugarcane: with or without burning. Unburnt sugarcane harvesting was more common in the cooler months, while the harvesting of burnt sugarcane increased twofold during the hotter months, as described in a previous publication (Tantipanjaporn et al. 2025). All participants in this study performed manual sugarcane cutting. The participants’ harvesting tasks involved manually cutting sugarcane with a machete, removing the leaves, and piling the stalks in rows on the ground. However, approximately 6% of participants performed a combination of harvesting tasks with either high-physically active tasks (e.g., harvesting combined with levelling sugarcane on a truck) or low-physically active tasks (e.g., harvesting combined with counting or driving). Work tasks were similar for both men and women, and also for different age groups (Table S2 in Supplementary Information).

### Number of layers of shirt

Most of the participants wore two layers of shirt (73.2%), followed by one layer (22,4%). There was a significant difference in clothing habits between genders (p-value = 0.029). Females were more likely to wear multiple layers; specifically, 80.8% of women wore two layers, compared to 67.3% of men. There were no significant differences in clothing layers based on seasons, harvesting methods, work tasks, or age groups (Table S3 in supplementary information).

### Heat stress exposure

The mean measured WBGT was 28.9 ± 2.6 °C (27.5 ± 2.5 °C in cooler months vs 30.5 ± 1.5 °C in hotter months) (Table S4 in supplementary information). The mean Tdb was 31.8 ± 2.3 °C (30.5 ± 1.9 vs 33.6 ± 1.4), Tnwb was 24.2 ± 3.0 °C (22.7 ± 3.0 vs 26.1 ± 1.5), Tg was 42.6 ± 3.1 °C (41.7 ± 2.5 vs 43.7 ± 3.4), and Rh was 33.5 ± 7.9% (31.4 ± 8.5 vs 36.0 ± 6.5). After adding CAF to the measured WBGT for each person, the average WBGT_eff_-FS-TWA from both seasons was 31.3 ± 2.8 °C, with a range of 22.9 – 35.4 °C. The average WBGT_eff_-FS-TWA during the hotter month was approximately 33 °C, which was higher than the average of 30 °C recorded in the cooler months (Table 2).

**Table 2:** WBGT_eff_-FS-TWA results of the study participants

WBGT_eff_-FS-TWA (°C)	n (%)		
	Total (n = 295)	Cooler months (n = 148)	Hotter months (n = 147)
> 34.0 32.1 – 34.0 30.1 – 32.0 28.1 – 30.0 26.1 – 28.0 ≤ 26.0	29 (9.8) 128 (43.4) 37 (12.5) 62 (21.0) 24 (8.1) 15 (5.1)	0 (0) 44 (29.7) 16 (10.8) 53 (35.8) 20 (13.5) 15 (10.1)	29 (19.7) 84 (57.1) 21 (14.3) 9 (6.1) 4 (2.7) 0 (0)
Mean ± S.D Range	31.3 ± 2.8 22.9 – 35.4	29.8 ± 2.7 22.9 – 33.9	32.8 ± 2.0 26.0 – 35.4

WBGT_eff_-FS-TWA = full work shift time-weighted average effective wet bulb globe temperature

### Heat-related symptoms of the participants over the previous 7 days

Table 3 shows the specific heat-related symptoms experienced by participants in the previous 7 days. A very high proportion of the participants (98.3%) experienced at least one heat related symptom, while 73.9% reported experiencing one or more severe heat-related symptoms. The top five heat-related symptoms were heavy sweating (86.1%), weakness/fatigue (71.2%), thirst (64.4%), elevated body temperature (58.6%), and rapid pulse (45.5%). The average number of overall symptoms and severe heat-related symptoms was 7.0 ± 3.9 out of 21 and 1.9 ± 1.8 out of 8, respectively (Figures S4 and S5 in Supplementary Information).

**Table 3:** Heat-related symptoms experienced by participants over the previous 7 days (n = 295)

No	Symptom	Yes; n (%)
1	Heavy sweating	254 (86.1)
2	Weakness/fatigue	210 (71.2)
3	Thirst	193 (65.4)
4	Elevated body temperature	173 (58.6)
5	Rapid pulse^c^	134 (45.4)
6	Dry mouth^a^	123 (41.8)
7	Headache^c^	110 (37.3)
8	Muscle cramps^a^	101 (34.4)
9	Dry and cracking skin^c^	94 (31.9)
10	Irritability	93 (31.5)
11	Rash on skin	91 (30.8)
12	Dizziness^b,c^	78 (26.7)
13	Very high body temperature or fever^c^	78 (26.4)
14	Difficulty in breathing	77 (26.1)
15	Urogenital issues such as oliguria or dysuria	55 (18.6)
16	Fainting	45 (15.3)
17	Confusion^c^	43 (14.6)
18	Swelling hands or feet	37 (12.5)
19	Nausea or vomiting^c^	33 (11.2)
20	Blisters on skin	31 (10.5)
21	Unconsciousness^a,c^	3 (1.0)

^a^1 Case of missing data

^b^3 Cases of missing data

^c^Severe heat-related symptoms

### Effects of heat stress exposure on heat-related symptoms

After adjusting for all potential confounders, heat stress exposure (WBGT_eff_-FS-TWA) was observed to be associated with an increased risk of heat-related symptoms (Table 4). In regard to the overall heat-related symptoms, the multivariable negative binomial regression model suggested that the RR associated with the number of heat-related symptoms due to a one-degree Celsius (C) increase in WBGT_eff_-FS-TWA was 1.10 (95% CI: 1.08, 1.13). This indicates that as WBGT_eff_-FS-TWA increases by 1 °C, the expected number of heat symptoms increases by 10%. In the secondary analyses for overall heat-related symptoms, the logistic regression model suggested the adjusted OR for heat symptoms was 1.47 (1.31, 1.66) for a one-degree increase in WBGT_eff_-FS-TWA for the median cut-off point and 1.50 (1.29, 1.75) for the 75th percentile cut-off point.

**Table 4:** Relative risks (RR) and Odds ratios (OR) for heat-related symptoms

Model	Explanatory variable	Overall heat-related symptoms			Severe heat-related symptoms		
		Symptom number	< / > Median	< / > 75th percentile	Symptom number	< / > Median	< / > 75th percentile
		RR (95% CI)	OR (95% CI)	OR (95% CI)	RR (95% CI)	OR (95% CI)	OR (95% CI)
Unadjusted	WBGT_eff_-FS-TWA	1.12 (1.09, 1.14)	1.47 (1.33, 1.64)	1.47 (1.29, 1.67)	1.21 (1.16, 1.25)	1.48 (1.33, 1.64)	1.49 (1.32, 1.68)
Adjusted^a^	WBGT_eff_-FS-TWA	1.10 (1.08, 1.13)	1.47 (1.31, 1.66)	1.50 (1.29, 1.75)	1.19 (1.14, 1.24)	1.57 (1.37, 1.79)	1.49 (1.30, 1.72)

WBGT_eff_-FS-TWA = full work shift time-weighted average effective Wet Bulb Globe Temperature

RR = Relative risk; OR = Odds ratios

^a^adjusted for *socio-demographic variable group*: gender, age, education, and work experience; *health metrics variable group*: BMI and medical conditions; *health behaviour variable group*: alcohol drinking, smoking, caffeine intake, and average sleep duration; *mitigating variable group*: total duration breaks, total fluid intake at work, and hours worked per week; and *working task group*: main task in the previous 7 days

In the case of heat severe heat-related symptoms, the adjusted RR of the number of severe heat-related symptoms was 1.19 (1.14, 1.24), reflecting a 19% higher risk of greater number of severe heat-related symptoms for a one-degree increase in WBGT_eff_-FS-TWA. In the secondary analysis of severe symptoms, the adjusted ORs for every one degree increase in WBGT_eff_-FS-TWA were 1.57 (1.37, 1.79) for the median cut-off point and 1.49 (1.30, 1.72) for the 75^th^ percentile cut-off.

Across all models for both overall and severe heat-related symptoms, the interaction between heat stress exposure (WBGT_eff_-FS-TWA) and gender was not statistically significant. This indicates no strong evidence of a difference in the exposure effect between males and females. While the point estimates suggest that the risk or odds of symptoms associated with heat stress exposure were slightly lower for females compared to males, these differences were not significant (Table S5).

Detailed full models of adjusted confounding variables for the overall heat-related symptoms and the severe heat-related symptoms are provided in Tables S6 and S7 (Supplementary Information), respectively. This study also analysed the data using the full work shift time-weighted average measured WBGT (WBGT_meas_-FS-TWA) that did not incorporate a CAF. These results are presented in Supplementary Table S8. Overall, the models using measured WBGT provided similar results to the effective WBGT models, showing that heat exposure remains significantly associated with an increased risk of heat-related symptoms regardless of the CAF application. The results of the investigation of non-linear relationships using piecewise linear models at different cut-off points, 30 °C, 29 °C, and 28 °C, indicated that there is no evidence for non-linearity (Table S9, Supplementary Information). The WBGT_eff_-FS-TWA categorical data revealed that there were clear linear trends, with heat-related symptoms increasing consistently across the WBGT_eff_-FS-TWA ranges (Table S10, Supplementary Information). Additionally, the average number of heat symptoms in the cooler months (6.8 ± 0.4 out of 21) was slightly lower than in the hotter month (7.11 ± 3.9), but this difference was not statistically significant.

## Discussion

This study determined the effects of heat stress exposure in Thai sugarcane workers, represented as WBGT_eff_-FS-TWA, on heat-related symptoms in the previous 7 days, with a focus on overall heat symptoms and severe heat-related symptoms. A high prevalence of heat-related symptoms in the previous 7 days was found. Heat stress exposure was significantly associated with an increased risk of both overall heat-related symptoms and severe heat-related symptoms. This highlights the severity of heat stress exposure and its impact on workers’ health.

The heat-related symptoms observed in this study were characteristic of acute heat-related illnesses, defined as a spectrum ranging from minor illnesses (i.e. heat rash, edema, syncope, cramps, and tetany) to potentially life-threatening major illnesses (i.e. heat exhaustion, and heat strokes) (Grubenhoff et al. 2007; Thawillarp et al. 2015). Minard and Copman (1963, as cited in National Institute for Occupational Safety and Health 2016) found that even though heat illnesses are interlinked, each has its own clinical characteristics.

The prevalence of heat-related symptoms of the workers in this study was very high. Nearly all participants experienced at least one heat-related symptom, with a considerable proportion (74%) suffering from at least one severe heat-related symptom. The heat symptom results in the study were consistent with a study by Venugopal et al. (2015), who reported that 96% of agricultural workers in India had at least one heat-related symptom, out of a total of 10 symptoms identified in their research. The symptoms reported in Venugopal et al.’s study were largely similar to those in this study. The main difference is how severe heat-related symptoms or heat stroke was handled. In their study, heat stroke was considered as a single symptom. In the current study, heat stroke was instead treated as a heat-related illness, and participants were asked about specific severe symptoms characteristic of heat stroke. However, the prevalence of heat-related symptoms in this study seemed to be higher than most previous studies on farmworkers in the United States (Kearney et al. 2016; Mutic et al. 2018; Smith et al. 2021) and Thailand (Kiatkitroj et al. 2022; Wichatorn & Chaiklieng 2022b). These differences might depend on the number of studied symptoms, the type of work, the agricultural season, the symptom reporting period, the local climate, or yearly increase in temperature. The most common heat symptoms reported in this study were heavy sweating (86.1%), weakness/fatigue (71.2%), and thirst (64.4%). This was similar to previous studies in Thailand (Boonruksa et al. 2020; Kiatkitroj et al. 2022; Langkulsen & Vichit-Vadakan 2018), Cambodia (Radir et al. 2017), the United States (Mutic et al. 2018), Slovenia (Pogačar et al. 2017), and Nigeria (Sadiq et al. 2019).

There were eight severe heat-related symptoms characteristic of heat stroke in this study. However, some of these including dizziness, headache, and nausea or vomiting can also be classified as symptoms of heat exhaustion. In this study, they were grouped under severe heat-related symptoms or heat stroke because they often precede sustained loss of consciousness which plays a critical role in the progression to heat stroke (National Institute for Occupational Safety and Health 2016). Notably, heat stroke often begins suddenly without warning signs, but in some cases, it can develop gradually over several hours or, rarely, days with nonspecific symptoms such as dizziness, headache, and nausea or vomiting (Leon & Bouchama 2015). Additionally, classic heat stroke, which results from heat exposure, is also characterised by neurological dysfunction, including headache, delirium, convulsions and coma (Di Lorenzo et al. 2009). Therefore, classifying headache under heat stroke helps ensure timely identification of extreme heat stress and reduce the risk of underestimating neurological involvement. While, symptoms like headache and nausea or vomiting can stem from yet other conditions (Spector et al. 2015), participants in the current study were specifically asked whether they had experienced these symptoms during work in the 7 days prior to the data collection day.

None of the previous studies appear to specifically mention symptoms characteristic of heat stroke. The most commonly used definition of heat stroke worldwide is Bouchama’s definition (Hifumi et al. 2018). Heat stroke is defined clinically as a core body temperature that rises above 40 °C and that is accompanied by hot, dry skin and central nervous system abnormalities such as delirium, convulsions, or coma (Bouchama & Knochel 2002). Most previous studies examined overall heat-related symptoms, not only symptoms of heat stroke (Arnold et al. 2020; Boonruksa et al. 2020; Kiatkitroj et al. 2022; Langkulsen & Vichit-Vadakan 2018; Mutic et al. 2018; Pogačar et al. 2017; Radir et al. 2017; Sadiq et al. 2019; Venugopal et al. 2015). Thus, there have been no previous studies with which to compare the severe symptoms associated with heat stroke data from this study. Therefore, our results provide a new and important finding for this field. Future research should continue to focus on these severe symptoms to better protect workers from the most dangerous effects of heat.

Heat stress exposure was associated with an increased risk of heat-related symptoms, following adjustment for potential confounding factors. The results of the primary analysis using a negative binomial model showed that as WBGT_eff_-FS-TWA increased by one degree Celsius, the risk of greater number of overall heat symptoms increased by 10% and a notably higher 19% increase in the odds of more severe heat-related symptoms. The higher association with severe heat-related symptoms might reflect their greater sensitivity to extreme heat because these symptoms normally happen at high levels of heat stress. The count distribution is generally assumed to be able to reach an infinite number of counts, however the count data in this study had a much smaller range, with only 8 severe heat-related symptoms and 21 for overall symptoms. While this study was unable to fit a truncated Negative Binomial model, this limitation is not anticipated to have a significant impact on the main findings.

In the secondary analyses, as WBGT_eff_-FS-TWA increased by one-degree, the odds of overall heat symptoms and severe heat-related symptoms increased by nearly 50% for both the 75^th^ percentile cut-off point (severe cases) and the median cut-off point (moderately severe cases). A study of Thai construction workers and foundry workers showed that the adjusted OR of for every one-degree Celsius increase in WBGT, an OR of having heat symptoms was1.85 (95% CI: 1.41–2.46) (Phanprasit et al. 2021). Melaku et al. (2024) studied hospitality business kitchen workers in Ethiopia and found that WBGT (based on hygrometer readings) above Threshold Limit Value (TLV) (Adjusted OR: 2.15; 95% CI (1.35, 3.42)) was significantly related to increased prevalence of heat-related symptoms. A study among Coast Guard Deepwater Horizon disaster responders in the United States by Erickson et al. (2019) reported that participants with the highest WBGT category had significantly increased prevalence of heat symptoms, compared to those in the lowest category (Prevalence ratios = 2.22 (95% CI: 1.61, 3.06)). When comparing our findings to previous literature, it is important to note that many previous studies rely solely on measured WBGT without a clothing factor (Erickson et al. 2019; Melaku et al. 2024; Phanprasit et al. 2021). Our supplementary analysis (Tables S8) confirms that even without applying the CAF, the significant association between heat exposure (measured WBGT) and heat-related symptoms remains in this population. This highlights the significant environmental heat burden experienced by these workers.

The adjusted OR/RR for the effect of heat stress exposure on heat symptoms in this study, therefore seemed to be different from previous studies (Erickson et al. 2019; Melaku et al. 2024; Phanprasit et al. 2021). This difference could possibly be explained by four factors. First, this study assessed a higher number of heat symptoms, totalling 21, compared to the 8 symptoms studied by Phanprasit et al. (2021), 7 symptoms by Melaku et al. (2024), and 5 symptoms by Erickson et al. (2019). Second, there were differences in determining the cut-off points for the symptoms. For example, previous studies defined the presence of heat-related symptoms as reporting at least one symptom in their analysis (Erickson et al. 2019; Melaku et al. 2024; Phanprasit et al. 2021). The time frames for reporting symptoms also differed; for example, Melaku et al. (2024) asked whether workers had experienced heat-related symptoms in the past six months, while Phanprasit et al. (2021) asked whether symptoms occurred during work in a hot environment. Third, the physical workload was different from the other studies, since the participants in this study had a high workload. The workloads of Thai construction workers and foundry workers, as reported by Phanprasit et al. (2021), and hospitality business kitchen workers, as reported by Melaku et al. (2024) ranged from light to heavy depending on the specific tasks. Both studies accounted for workload in their analyses of heat stress and related symptoms. In contrast, Erickson et al. (2019) did not assess workload among their participants. Finally, most of the participants in this study were exposed to very high levels of heat stress exposure, because they worked outdoors all day in a hotter environment.

Additionally, although the difference in the number of symptoms between seasons was not statistically significant, symptoms tended to be slightly more frequent in the hotter month. This is possibly because collecting data in the cooler months was close to the hot month, when temperatures were already rising. This may not accurately represent the entire cool season (mid-October to mid-February), since temperatures tend to be lower in December and January.

The mean dry-bulb temperature (air temperature) during the cooler months was 30.5 °C (mean relative humidity: 31.4%), based on data collected over 14 non-consecutive days from January 18 to February 10. During the hotter month, the mean dry-bulb temperature was 33.6 °C (mean relative humidity: 36.0%), based on data collected over 11 days from March 11 to March 20. The mean temperatures in both seasons were slightly higher than the corresponding averages for the central region of Thailand, where the Thai Meteorological Department classifies Nakhon Sawan Province (the study site). Regional averages range from 27.7 °C (January) to 29.5 °C (February) for the cool months and 31.0 °C (March) for the hot month (Thai Meteorological Department, 2024). This difference may be due to Nakhon Sawan, the study area, being one of the hottest provinces in Thailand, with a new March temperature record of 41.4 °C in 2024 (Thai Meteorological Department, 2024), which raised the central region’s average temperature. In addition, the cooler-month data in this study were collected toward the end of the season, when temperatures were rising. Thailand is located in the tropics near the equator, resulting in hot and humid conditions for most of the year. However, the relative humidity reported in this study was lower than the regional averages of 71% in the cool season and 69% in the hot season (Thai Meteorological Department, n.d .^2024^). This could be because, in inland areas, particularly from the central region northward, relative humidity decreases noticeably during the cool and hot seasons (Thai Meteorological Department, n.d. 2024).

There are several strengths and limitations in this cross-sectional study. One strength is that the study utilized multiple forms of analysis to determine the effects of heat stress on heat symptoms, and the different analyses demonstrated consistency across results. Methodologically, heat exposure assessments were conducted throughout the work shift rather than relying on point-in-time measurements or ambient weather station data, which is a highly robust approach. Furthermore, participants’ clothing was recorded to determine the appropriate CAF, which added to the measured WBGT to calculate the effective WBGT. Another strength is that this study specifically tested the effects of exposure to heat stress on severe heat-related symptoms characteristic of heat stroke, which is not reported in previous studies. Heat stroke represents the most severe and life-threatening effect of heat exposure, so studying its symptoms specifically is important. Also, gathering data using a questionnaire survey instead of relying on past medical records made it possible to more fully record participants’ symptoms related to heat. However, the use of a questionnaire to determine the symptoms is also a limitation as it is based on self-reporting and thus the participants’ perception, which could vary greatly between individuals. Also, some heat-related symptoms can be linked to other conditions. Since this was a cross-sectional design, it limited the ability to evaluate the temporality of exposures and outcomes. Another potential limitation is that all symptoms were considered as the same level of severity, but in reality, some symptoms (such as, unconsciousness, confusion or dry and cracking skin) linked to heat stroke are more tightly than others. The inability to fully characterise heat stroke symptoms appeared to be a limitation of this study and should be a key focus for future research. There is also lack of a standardised, harmonised questionnaire for assessing heat-related and heat stroke symptoms. In addition, there is currently no agreed weighting of symptoms by severity, which limits comparability across studies. Finally, the participants were asked whether they had experienced any symptoms in the 7 days prior to the day of heat measurement. This suggests the possibility of selection bias, and the healthy worker effect, where the presence of symptoms may have affected participation. For example, workers who experienced severe heat-related symptoms may have been absent from work or reluctant to participate. This could result in a possible underrepresentation of those who are more negatively affected, causing an underestimation of the association between heat stress exposure and heat-related symptoms. However, data collection was done over 25 days in two seasons. Given that some workers who were absent early in the study might have returned and been included later, this might have helped to reduce this risk. Although the incorporation of CAF into the analysis in this study is one of its novelties and strengths, it also requires deeper consideration of the findings. While workers wore only one layer of trousers, they typically wore multiple upper-body layers for protection against heat and plant debris which is a traditional practice in Thai agriculture. We applied a CAF of 3 °C based on ACGIH standards for these woven layers. However, this may overestimate the total physiological heat stress because it does not fully account for heat loss from the lower body. Despite this potential overestimation compared to the values suggested by Bernard et al. (2005), our calculation provides a precautionary estimate that prioritizes worker safety.

## Conclusion

With an increase in average global temperatures and an increase in the frequency and intensity of heatwaves due to climate change, the severity of heat stress exposure among outdoor workers is also rising. Monitoring and understanding the current impact of changing temperatures on heat-related symptoms is vital for ensuring the health and safety of outdoor workers and implementing effective preventive measures. The results of this study showed very high prevalence of heat-related symptoms experienced by Thai sugarcane workers in the past 7 days during harvesting season. The heat stress exposure, measured as WBGT_eff_-FS-TWA was significantly related to an increased risk of heat-related and severe heat-related symptoms. Given the urgency of reducing heat stress, these updated data can be valuable for policymakers in developing effective heat-related health policies for outdoor informal workers.

## Supplementary Information

Below is the link to the electronic supplementary material.

Supplementary Material 1

## Data Availability

The data that support the findings of this study are available from the corresponding author, upon reasonable request.
