# Supplementary material for: Occupational related heat stress exposure and heat-related symptoms among sugarcane workers in Thailand
Source: Int Arch Occup Environ Health. 2026 Jun 16;99(5):29. doi: 10.1007/s00420-026-02216-4 (PMC13272210; doi:10.1007/s00420-026-02216-4)
Supplement: Supplementary file 1 — Supplementary Material 1 [file 420_2026_2216_MOESM1_ESM.docx]

Supplementary Information

Occupational Related Heat Stress Exposure and Heat-Related Symptoms Among Sugarcane Workers in Thailand

Tadpong Tantipanjaporn^1*^, Andrew Povey^2^, Holly A. Shiels^3^, Matthew Gittins^4^, Martie van Tongeren^2,5^

^1^ Division of Occupational Health and Safety, Faculty of Public Health, Naresuan University, 99 Moo 9, Thapo Sub-district, Muang District, Phitsanulok City, 65000, Thailand

^2^ Centre for Occupational and Environmental Health, School of Health Sciences, Faculty of Biology, Medicine and Health, University of Manchester, Ellen Wilkinson Building (Block C), Oxford Road, Manchester, M13 9PL, United Kingdom

^3^ Division of Cardiovascular Sciences, School of Medical Sciences, Faculty of Biology, Medicine and Health, University of Manchester, Core Technology Facility, 46 Grafton Street, Manchester, M13 9NT, United Kingdom

^4^ Centre for Biostatistics, School of Health Sciences, Faculty of Biology, Medicine and Health, University of Manchester, Jean McFarlane Building, Oxford Road, Manchester, M13 9PL, United Kingdom

^5^ Thomas Ashton Institute for Risk and Regulatory Research, The University of Manchester, Nancy Rothwell Building, Oxford Road, Manchester, M13 9PL, United Kingdom

*Corresponding author: Email: tadpongt@nu.ac.th


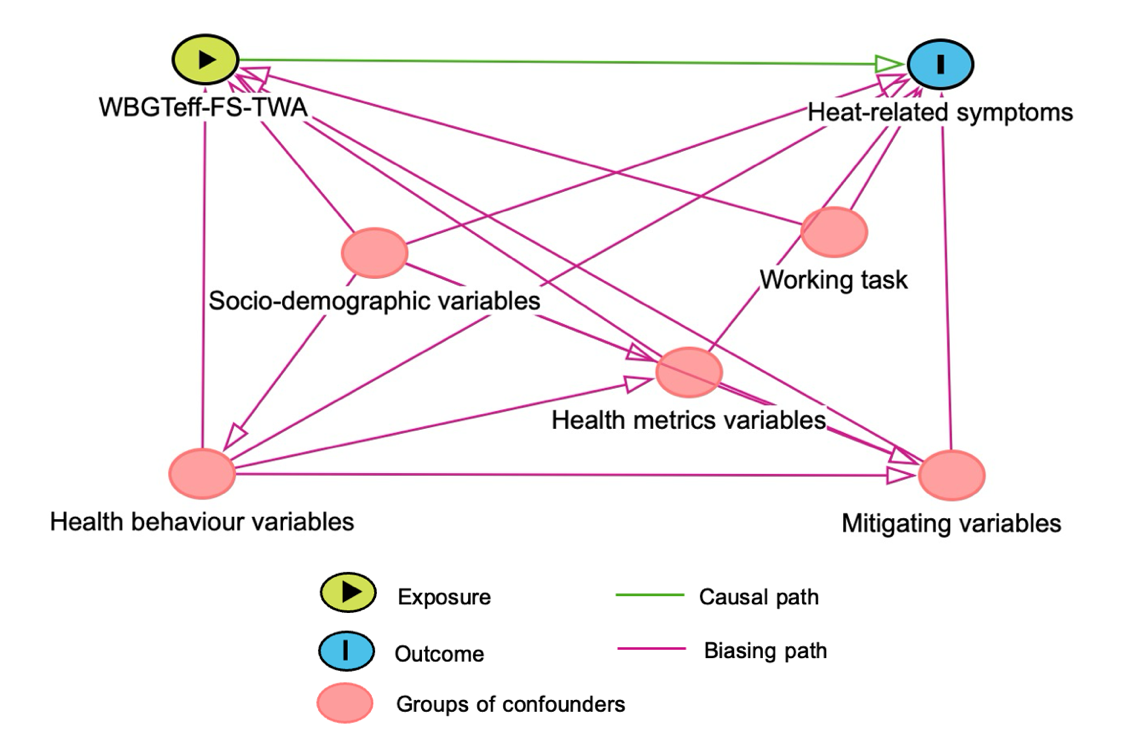


Figure S1 A Directed acyclic graph (DAG) for hypothetical causal relationships between the primary explanatory variables (a full work shift time-weighted average effective Wet Bulb Globe Temperature or WBGT_eff_-FS-TWA) and the outcome variable (heat-related symptoms), along with groups of confounders

*Socio-demographic variable group*: gender, age, education, and work experience; *health metrics variable group*: BMI and medical conditions; *health behaviour variable group*: alcohol drinking, smoking, caffeine intake, and average sleep duration; *mitigating variable group*: total duration breaks, total fluid intake at work, and hours worked per week; and *working task group*: main task in the previous 7 days

In our adjusted models (Figure S1), health behavioural, mitigating and working variables such as alcohol consumption, smoking, caffeine intake, average sleep duration, total break duration, total fluid intake at work, weekly working hours, and main task in the previous 7 days were treated as confounders. These baseline 7-day habits significantly influence a worker’s behavioural interaction with the environment (e.g., by altering work duration, physical exertion, or resting patterns), thereby modifying their individual WBGT_eff_-FS-TWA.


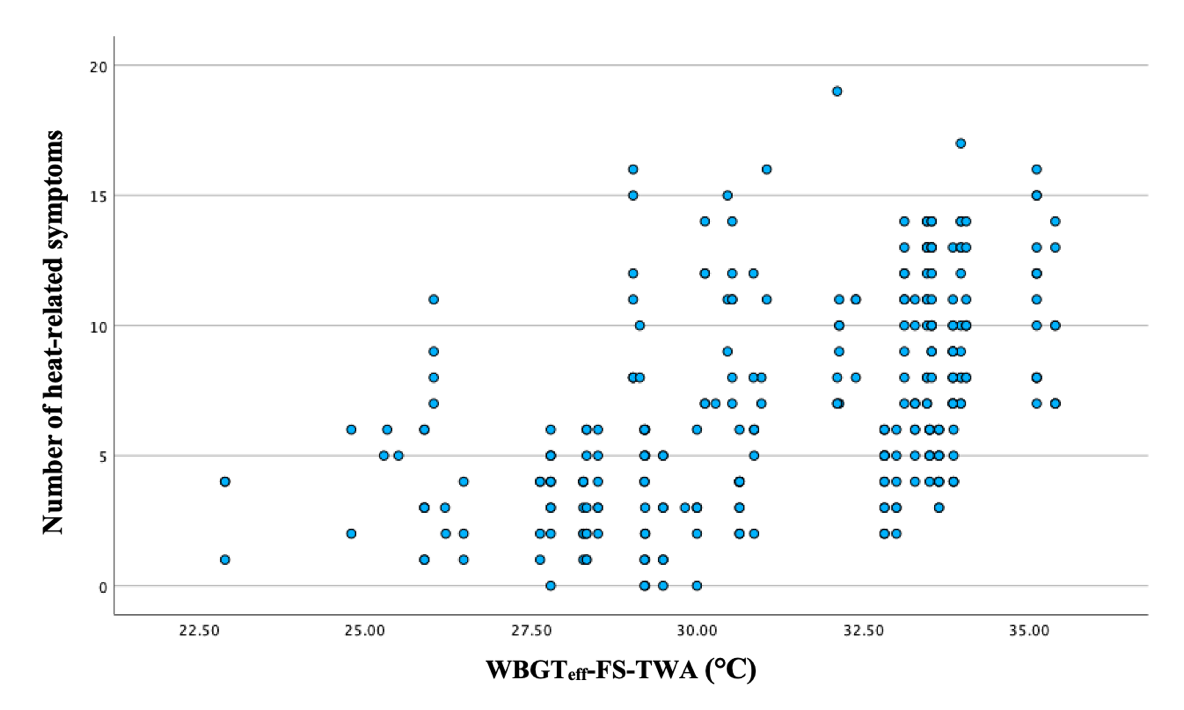


Figure S2 A scatter plot of full work shift time-weighted average effective Wet Bulb Globe Temperature (WBGT_eff_-FS-TWA) and the number of overall heat-related symptoms (n = 295)


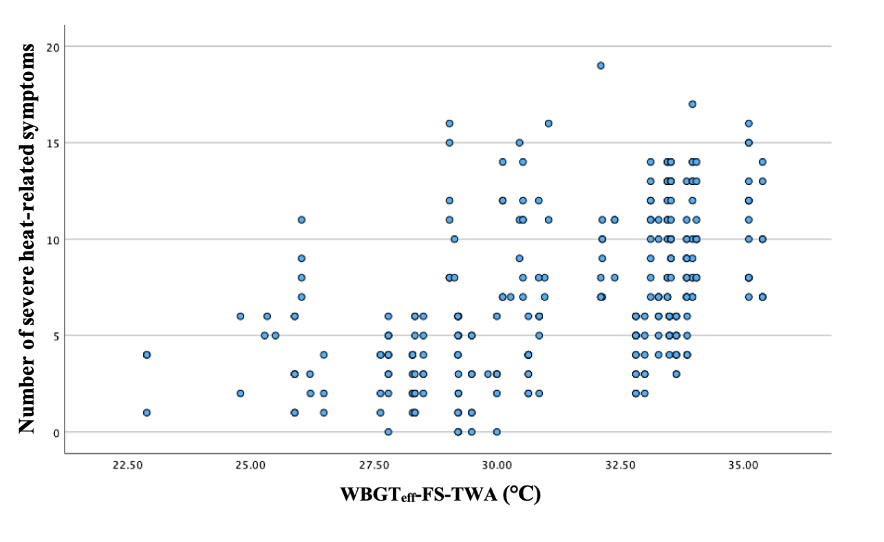


Figure S3 A scatter plot of full work shift time-weighted average effective Wet Bulb Globe Temperature (WBGT_eff_-FS-TWA) and the number of severe heat-related symptoms associated with heat stroke (n = 295)

Figure S4 The number of overall heat-related symptoms experienced by participants in the last 7 days

Figure S5 The number of severe heat-related symptoms associated with heat stroke experienced by participants in the last 7 days

Table S1 Summary results from additional models exploring the effect of specific groups of confounders in a step-by-step process: relative risk (95% CI) and odds ratio (95% CI) of overall heat-related symptoms and severe heat-related symptoms

| Explanatory variable | Model | Overall heat-related symptoms | | | Severe heat-related symptoms | | |
| --- | --- | --- | --- | --- | --- | --- | --- |
|  |  | Symptom number | </>Median | </>75^th^ percentile | Symptom number | </>Median | </>75^th^ percentile |
|  |  | RR (95% CI)^†^ | OR (95% CI)^‡^ | OR (95% CI)^‡^ | RR (95% CI)^†^ | OR (95% CI)^‡^ | OR (95% CI)^‡^ |
| WBGT_eff_-FS-TWA | 0 | 1.12 (1.09 - 1.14) | 1.47 (1.33 - 1.64) | 1.47 (1.29 - 1.67) | 1.21 (1.16 - 1.25) | 1.48 (1.33 - 1.64) | 1.49 (1.32 - 1.68) |
|  | 1 | 1.11 (1.09 - 1.13) | 1.46 (1.31 - 1.62) | 1.47 (1.28 - 1.69) | 1.19 (1.15 - 1.24) | 1.48 (1.32 - 1.65) | 1.47 (1.30 - 1.67) |
|  | 2 | 1.11 (1.09 - 1.13) | 1.49 (1.33 - 1.67) | 1.49 (1.29 - 1.73) | 1.19 (1.15 - 1.24) | 1.49 (1.32 - 1.67) | 1.48 (1.30 - 1.68) |
|  | 3 | 1.11 (1.09 - 1.14) | 1.50 (1.33 - 1.68) | 1.53 (1.32 - 1.78) | 1.20 (1.15 - 1.25) | 1.54 (1.35 - 1.74) | 1.52 (1.33 - 1.75) |
|  | 4 | 1.10 (1.08 - 1.13) | 1.48 (1.31 - 1.67) | 1.50 (1.29 - 1.75) | 1.19 (1.14 - 1.24) | 1.52 (1.34 - 1.73) | 1.50 (1.30 - 1.72) |
|  | 5 | 1.10 (1.08 - 1.13) | 1.47 (1.31 - 1.66) | 1.50 (1.29 - 1.75) | 1.19 (1.14 - 1.24) | 1.57 (1.37 - 1.79) | 1.49 (1.30 - 1.72) |

WBGT_eff_-FS-TWA = full work shift time-weighted average effective Wet Bulb Globe Temperature

^†^Generalised linear negative binomial model; ^‡^Generalised binary logistic model

RR = Relative risk; OR = Odds ratios

Model 0: unadjusted

Model 1: adjusted for socio-demographic variables

Model 2: adjusted for socio-demographic variables, and health metrics variables

Model 3: adjusted for socio-demographic variables, health metrics variables, and health behaviour variables

Model 4: adjusted for socio-demographic variables, health metrics variables, health behaviour variables, and mitigating variables

Model 5: adjusted for socio-demographic variables, health metrics variables, health behaviour variables, mitigating variables, and the working task

Table S2 Distribution of work tasks by gender and age group (n=295)

| Characteristics | | n | Work Tasks^a^ | | p-value^b^ |
| --- | --- | --- | --- | --- | --- |
|  |  |  | Strenuous manual tasks  n(%) | Mixed or supportive tasks  n(%) |  |
| Gender | Male | 165 | 154 (93.3) | 11 (6.7) | 0.080 |
|  | Female | 130 | 127 (97.7) | 3 (2.3) |  |
| Age | ≤ 40 years | 133 | 126 (94.7) | 7 (5.3) | 0.705 |
|  | < 40 years | 162 | 155 (95.7) | 7 (4.3) |  |

^a^ Strenuous manual tasks: harvesting only or harvesting with additional high physically active levels of work, i.e. levelling sugarcane on a track

Mixed or supportive tasks: harvesting with additional low physically active levels of work, i.e. counting sugarcane, driving tractor, and driving sugarcane leaf remover

^b^ Chi-square test

Table S3 Distribution of number of shirt layers by seasons, harvesting methods, work tasks, gender and age group (n=295)

| Characteristics | | n | Number of shirt layers^c^ | | | p-value^d^ |
| --- | --- | --- | --- | --- | --- | --- |
|  |  |  | 1 layer; n(%)  (n=66; 22.4%) | 2 layers; n(%)  (n=216; 73.2%) | 3 layers; n(%)  (n=13; 4.4%) |  |
| Seasons | Cooler month | 148 | 30 (20.3) | 110 (74.3) | 8 (5.4) | 0.520 |
|  | Hotter month | 147 | 36 (24.5) | 106 (72.1) | 5 (3.4) |  |
| Harvesting methods | Burnt sugarcane | 117 | 21 (17.9) | 90 (76.9) | 6 (5.1) | 0.683 |
|  | Unburnt sugarcane | 103 | 26 (25.2) | 73 (70.9) | 4 (3.9) |  |
|  | Mixed harvesting^b^ | 75 | 19 (25.3) | 53 (70.7) | 3 (4.0) |  |
| Work Tasks^a^ | Strenuous manual tasks | 281 | 65 (23.1) | 204 (72.6) | 12 (4.3) | 0.352 |
|  | Mixed or supportive tasks | 14 | 1 (7.1) | 12 (85.3) | 1 (7.1) |  |
| Gender | Male | 165 | 46 (27.9) | 111 (67.3) | 8 (4.8) | 0.029* |
|  | Female | 130 | 20 (15.4) | 105 (80.8) | 5 (3.8) |  |
| Age | ≤ 40 years | 133 | 30 (22.6) | 100 (75.2) | 3 (2.3) | 0.263 |
|  | < 40 years | 162 | 36 (22.2) | 116 (71.6) | 10 (6.2) |  |

^a^ Strenuous manual tasks: harvesting only or harvesting with additional high physical levels of work, i.e. levelling sugarcane on a track

Mixed or supportive tasks: harvesting with additional low physical levels of work, i.e. counting sugarcane, driving tractor, and driving sugarcane leaf remover

^b^ Harvesting both burnt and unburnt sugarcane in one day

^c^ Types of shirt: T-shirt, shirt, or jacket made of polyester, cotton/polyester blend, or denim; Additionally, 82% of participants wore one layer of trousers, while 18% wore two layers.

^d^ Chi-square test *p-value < 0.05

Table S4 WBGT_meas_-FS-TWA results of the study participants

| WBGT_meas_-FS-TWA (°C) | n (%) | | |
| --- | --- | --- | --- |
|  | Total  (n = 295) | Cooler months  (n = 148) | Hotter months  (n = 147) |
| > 32.0  30.1 – 32.0  28.1 – 30.0  26.1 – 28.0  ≤ 26.0 | 28 (9.5)  134 (45.4)  34 (11.5)  40 (13.6)  59 (20.0) | 0 (0)  33 (22.3)  27 (18.2)  40 (27.0)  48 (32.4) | 28 (19.0)  101 (68.7)  7 (4.8)  0 (0)  11 (7.5) |
| Mean ± S.D.  Range | 28.9 ± 2.6  22.9 – 32.4 | 27.5 ± 2.5  22.9 – 30.6 | 30.5 ± 1.5  26.0 – 32.4 |

WBGT_meas_-FS-TWA = full work shift time-weighted average measured Wet Bulb Globe Temperature

Table S5 Interaction effect between WBGT_eff_-FS-TWA and gender

| Model | Explanatory variable | Overall heat-related symptoms | | | Severe heat-related symptoms | | |
| --- | --- | --- | --- | --- | --- | --- | --- |
|  |  | Symptom number | </>Median | </>75^th^ percentile | Symptom number | </>Median | </>75^th^ percentile |
|  |  | RR (95% CI) | OR (95% CI) | OR (95% CI) | RR (95% CI) | OR (95% CI) | OR (95% CI) |
| Adjusted model with the interaction term^a,b^ | WBGT_eff_-FS-TWA | 1.12 (1.08, 1.15) | 1.56 (1.33, 1.82) | 1.58 (1.25, 1.99) | 1.22 (1.16, 1.29) | 1.71 (1.42, 2.06) | 1.58 (1.29, 1.93) |
|  | Gender (Female vs Male) | 2.46 (0.60, 10.13) | 78.08 (0.05, 1.3x10^5^) | 29.50 (0.01, 5.7x10^5^) | 7.37 (0.5, 1x10^2^) | 414 (0.1, 1.2x10^6^) | 45 (0.01, 3.6x10^5^) |
|  | WBGT_eff_-FS-TWA*Gender | 0.98 (0.93, 1.02) | 0.98 (0.93, 1.02) | 0.92 (0.68, 1.24) | 0.94 (0.87, 1.02) | 0.83 (0.65, 1.07) | 0.88 (0.67, 1.16) |

WBGT_eff_-FS-TWA = full work shift time-weighted average effective Wet Bulb Globe Temperature

RR = Relative risk; OR = Odds ratios

^a^adjusted for *socio-demographic variable group*: gender, age, education, and work experience; *health metrics variable group*: BMI and medical conditions; *health behaviour variable group*: alcohol drinking, smoking, caffeine intake, and average sleep duration; *mitigating variable group*: total duration breaks, total fluid intake at work, and hours worked per week; and *working task group*: main task in the previous 7 days

^b^adjusted model included the interaction term between WBGT_eff_-FS-TWA and gender; Gender coded as 0 = male, 1 = female

Gender effect estimates are calculated at a WBGT of 0 °C. Because our data range is 22.9-35.4 °C, the lack of observations near zero results in the observed wide confidence intervals. Interpretation should focus on the interaction term rather than the gender main effect.

Table S6 Adjusted relative risk (95% CI) and odds ratio (95% CI) of overall heat-related symptoms (adjusted models)

| Explanatory variable | n | Overall heat-related symptoms | | |
| --- | --- | --- | --- | --- |
|  |  | Symptom number | </>Median | </>75^th^ percentile |
|  |  | Adj RR (95% CI) | Adj OR (95% CI) | Adj OR (95% CI) |
| WBGT_eff_-FS-TWA (°C) | 295 | 1.10 (1.08 - 1.13) | 1.47 (1.31 - 1.66) | 1.50 (1.29 - 1.75) |
| Gender |  |  |  |  |
| Female | 130 | 1.10 (0.94 - 1.26) | 0.94 (0.43 - 2.04) | 1.72 (0.75 - 3.93) |
| Male | 165 |  |  |  |
| Age (years) |  |  |  |  |
| ≥ 50 | 87 | 0.85 (0.72 - 1.01) | 0.50 (0.20 - 1.24) | 0.49 (0.18 - 1.29) |
| 40-49 | 84 | 0.88 (0.74 - 1.03) | 0.56 (0.23 - 1.38) | 0.53 (0.21 - 1.31) |
| 30-39 | 66 | 0.92 (0.78 - 1.08) | 0.71 (0.29 - 1.78) | 0.89 (0.36 - 2.20) |
| < 30 | 58 |  |  |  |
| Education |  |  |  |  |
| ≥ High school | 31 | 0.93 (0.77 - 1.12) | 0.77 (0.28 - 2.09) | 0.40 (0.13 - 1.20) |
| Middle school | 65 | 0.98 (0.84 - 1.13) | 1.03 (0.45 - 2.35) | 0.65 (0.28 - 1.49) |
| ≤ Primary school | 199 |  |  |  |
| Work experience (years) |  |  |  |  |
| > 10 | 59 | 0.93 (0.80 - 1.09) | 0.44 (0.20 - 0.99) | 0.72 (0.31 - 1.67) |
| 5.01-10 | 81 | 0.91 (0.80 - 1.04) | 0.52 (0.26 - 1.04) | 0.64 (0.31 - 1.34) |
| ≤ 5 | 155 |  |  |  |
| BMI |  |  |  |  |
| Obese I | 80 | 1.02 (0.89 - 1.17) | 1.95 (0.92 - 4.11) | 1.15 (0.54 - 2.44) |
| Overweight | 39 | 0.86 (0.72 - 1.02) | 0.84 (0.35 - 2.03) | 0.57 (0.20 - 1.59) |
| Underweight | 35 | 1.00 (0.84 - 1.20) | 0.74 (0.29 - 1.93) | 0.55 (0.20 - 1.57) |
| Normal weight | 141 |  |  |  |
| Medical conditions |  |  |  |  |
| Yes | 54 | 1.24 (1.07 - 1.43) | 3.73 (1.54 - 9.05) | 2.40 (1.12 - 5.13) |
| No | 241 |  |  |  |
| Alcohol drinking |  |  |  |  |
| Yes | 152 | 1.10 (0.98 - 1.24) | 0.95 (0.50 - 1.81) | 1.45 (0.73 - 2.88) |
| No | 143 |  |  |  |
| Smoking |  |  |  |  |
| Yes | 141 | 0.90 (0.78 - 1.04) | 0.77 (0.36 - 1.64) | 0.54 (0.23 - 1.28) |
| No | 154 |  |  |  |
| Caffeine intake |  |  |  |  |
| Yes | 283 | 1.08 (0.79 - 1.46) | 1.24 (0.29 - 5.29) | 3.46 (0.33 - 35.95) |
| No | 12 |  |  |  |
| Sleep duration (hours) | 295 | 0.99 (0.95 - 1.03) | 1.00 (0.80 - 1.25) | 0.83 (0.66 - 1.06) |
| Total duration breaks (hours) | 295 | 1.10 (1.03 - 1.18) | 1.11 (0.94 - 1.32) | 1.07 (0.89 - 1.28) |
| Total fluid intake at work (litres) | 295 | 1.02 (0.99 - 1.05) | 1.33 (0.92 - 1.92) | 1.46 (1.02 - 2.09) |
| Hour worked per week (hours) | 295 | 1.00 (1.00 - 1.01) | 1.01 (0.98 - 1.04) | 1.01 (0.98 - 1.04) |
| Main responsibility in the previous 7 days | | |  |  |
| Harvesting with a low-physical-level task | 14 | 0.91 (0.69 - 1.21) | 0.56 (0.15 - 2.09) | 0.48 (0.08 - 2.87) |
| Harvesting only/harvesting with a high-physical-level task | 281 |  |  |  |

WBGT_eff_-FS-TWA = full work shift time-weighted average effective Wet Bulb Globe Temperature; RR = Relative risk; OR = Odds ratios

Table S7 Adjusted relative risk (95% CI) and odds ratio (95% CI) of severe heat-related symptoms (adjusted models)

| Explanatory variable | n | Severe heat-related symptoms | | |
| --- | --- | --- | --- | --- |
|  |  | Symptom number | </>Median | </>75^th^ percentile |
|  |  | Adj RR (95% CI) | Adj OR (95% CI) | Adj OR (95% CI) |
| WBGT_eff_-FS-TWA (°C) | 295 | 1.19 (1.14 - 1.24) | 1.57 (1.37 - 1.79) | 1.49 (1.30 - 1.72) |
| Gender |  |  |  |  |
| Female | 130 | 1.11 (0.87 - 1.41) | 1.28 (0.59 - 2.78) | 0.83 (0.38 - 1.81) |
| Male | 165 |  |  |  |
| Age (years) |  |  |  |  |
| ≥ 50 | 87 | 0.68 (0.51 - 0.90) | 0.36 (0.14 - 0.91) | 0.36 (0.14 - 0.92) |
| 40-49 | 84 | 0.79 (0.61 - 1.03) | 0.72 (0.30 - 1.75) | 0.46 (0.19 - 1.10) |
| 30-39 | 66 | 0.82 (0.63 - 1.07) | 0.65 (0.27 - 1.59) | 0.47 (0.20 - 1.14) |
| < 30 | 58 |  |  |  |
| Education |  |  |  |  |
| ≥ High school | 31 | 0.97 (0.71 - 1.30) | 0.82 (0.30 - 2.26) | 0.93 (0.34 - 2.55) |
| Middle school | 65 | 0.93 (0.73 - 1.18) | 0.72 (0.32 - 1.61) | 1.10 (0.50 - 2.43) |
| ≤ Primary school | 199 |  |  |  |
| Work experience (years) |  |  |  |  |
| > 10 | 59 | 1.13 (0.88 - 1.44) | 0.87 (0.38 - 1.96) | 1.55 (0.69 - 3.46) |
| 5.01-10 | 81 | 0.86 (0.69 - 1.07) | 0.46 (0.23 - 0.93) | 1.17 (0.58 - 2.33) |
| ≤ 5 | 155 |  |  |  |
| BMI |  |  |  |  |
| Obese I | 80 | 0.89 (0.71 - 1.11) | 0.99 (0.47 - 2.09) | 0.67 (0.32 - 1.39) |
| Overweight | 39 | 0.81 (0.60 - 1.09) | 0.72 (0.29 - 1.79) | 0.58 (0.22 - 1.49) |
| Underweight | 35 | 0.95 (0.71 - 1.27) | 0.71 (0.27 - 1.87) | 0.49 (0.19 - 1.27) |
| Normal weight | 141 |  |  |  |
| Medical conditions |  |  |  |  |
| Yes | 54 | 1.38 (1.10 - 1.73) | 3.94 (1.70 - 9.13) | 2.56 (1.21 - 5.42) |
| No | 241 |  |  |  |
| Alcohol drinking |  |  |  |  |
| Yes | 152 | 1.22 (1.00 - 1.49) | 1.52 (0.80 - 2.91) | 1.68 (0.87 - 3.25) |
| No | 143 |  |  |  |
| Smoking |  |  |  |  |
| Yes | 141 | 0.69 (0.55 - 0.88) | 0.37 (0.17 - 0.82) | 0.29 (0.13 - 0.64) |
| No | 154 |  |  |  |
| Caffeine intake |  |  |  |  |
| Yes | 283 | 1.08 (0.63 - 1.84) | 1.25 (0.27 - 5.84) | 1.69 (0.32 - 9.00) |
| No | 12 |  |  |  |
| Sleep duration (hours) | 295 | 0.99 (0.93 - 1.06) | 0.87 (0.70 - 1.10) | 1.06 (0.84 - 1.33) |
| Total duration breaks (hours) | 295 | 1.11 (1.00 - 1.23) | 1.01 (0.85 - 1.20) | 0.96 (0.81 - 1.15) |
| Total fluid intake at work (litres) | 295 | 1.00 (0.95 - 1.05) | 1.19 (0.83 - 1.70) | 1.55 (1.09 - 2.21) |
| Hour worked per week (hours) | 295 | 1.01 (1.00 - 1.01) | 1.02 (1.00 - 1.05) | 1.00 (0.98 - 1.03) |
| Main responsibility in the previous 7 days | | | | |
| Harvesting with a low-physical-level task | 14 | 1.32 (0.86 - 2.01) | 7.89 (1.82 - 34.10) | 0.40 (0.08 - 2.03) |
| Harvesting only/harvesting with a high-physical-level task | 281 |  |  |  |

WBGT_eff_-FS-TWA = full work shift time-weighted average effective Wet Bulb Globe Temperature; RR = Relative risk; OR = Odds ratios

Table S8 Relative risk (95% CI) from a generalised linear negative binomial model and odds ratios (95% CI) from a generalised binary logistic model of overall heat-related symptom and severe heat-related symptoms [Explanatory variable as measured WBGT]

| Model | Explanatory variable | Overall heat-related symptoms | | | Severe heat-related symptoms | | |
| --- | --- | --- | --- | --- | --- | --- | --- |
|  |  | Symptom number | </>Median | </>75^th^ percentile | Symptom number | </>Median | </>75^th^ percentile |
|  |  | RR (95% CI) | OR (95% CI) | OR (95% CI) | RR (95% CI) | OR (95% CI) | OR (95% CI) |
| Unadjusted | WBGT_meas_-FS-TWA | 1.15 (1.12, 1.17) | 1.60 (1.43, 1.80) | 1.66 (1.39, 1.98) | 1.27 (1.22, 1.34) | 1.62 (1.43, 1.84) | 1.71 (1.45, 2.02) |
| Adjusted^a^ | WBGT_meas_-FS-TWA | 1.13 (1.10, 1.16) | 1.59 (1.39, 1.81) | 1.68 (1.38, 2.05) | 1.25 (1.19, 1.31) | 1.65 (1.43, 1.92) | 1.69 (1.41, 2.03) |

WBGT_meas_-FS-TWA = full work shift time-weighted average measured Wet Bulb Globe Temperature

RR = Relative risk; OR = Odds ratios

^a^adjusted for *socio-demographic variable group*: gender, age, education, and work experience; *health metrics variable group*: BMI and medical conditions; *health behaviour variable group*: alcohol drinking, smoking, caffeine intake, and average sleep duration; *mitigating variable group*: total duration breaks, total fluid intake at work, and hours worked per week; and *working task group*: main task in the previous 7 days

Table S9 Investigation of non-linear relationships using piecewise linear models at cut-off points of WBGT_eff_-FS-TWA at 30, 29, and 28°C

| Explanatory variable | n | | B | | S.E. | | p-value | | Unadjusted RR/OR | | (95% CI) | | | AIC | BIC | |  |  |
| --- | --- | --- | --- | --- | --- | --- | --- | --- | --- | --- | --- | --- | --- | --- | --- | --- | --- | --- |
|  |  |  |  |  |  |  |  |  |  |  | Lower | | Upper |  | |  | |  |
| Number of overall heat-related symptoms^a^ | | | | | | | | | | | | | |  | |  | | |
| Single linear | | | | | | | | | | | | | |  | |  | | |
| WBGT_eff_-FS-TWA (°C) | | 295 | | 0.11 | | 0.01 | | <.001** | | 1.12 | | 1.09 | 1.14 | 1544.95 | | 1555.01 | |  |
| Piecewise linear at a cut-off point of 28 °C | | | | | | | | | | | | | |  | |  | | |
| ≤ 28 °C of WBGT | | 295 | | 0.02 | | 0.05 | | 0.740 | | 1.02 | | 0.92 | 1.12 | 1542.46 | | 1557.20 | |  |
| > 28 °C of WBGT | | 295 | | 0.13 | | 0.01 | | <.001** | | 1.13 | | 1.10 | 1.17 |  | |  | |  |
| Piecewise linear at a cut-off point of 29 °C | | | | | | | | | | | | | |  | |  | | |
| ≤ 29 °C of WBGT | | 295 | | 0.05 | | 0.04 | | 0.151 | | 1.05 | | 0.98 | 1.13 | 1546.86 | | 1561.61 | |  |
| > 29 °C of WBGT | | 295 | | 0.13 | | 0.02 | | <.001** | | 1.13 | | 1.10 | 1.17 |  | |  | |  |
| Piecewise linear at a cut-off point of 30 °C | | | | | | | | | | | | | |  | |  | | |
| ≤ 30 °C of WBGT | | 295 | | 0.10 | | 0.03 | | 0.001* | | 1.10 | | 1.04 | 1.17 | 1545.77 | | 1560.51 | |  |
| > 30 °C of WBGT | | 295 | | 0.12 | | 0.02 | | <.001** | | 1.12 | | 1.08 | 1.17 |  | |  | |  |
| Number of severe heat-related symptoms^a^ | | | | | | | | | | | | | |  | |  | | |
| Single linear | |  | |  | |  | |  | |  | |  |  |  | |  | |  |
| WBGT_eff_-FS-TWA (°C) | | 295 | | 0.19 | | 0.02 | | <.001** | | 1.21 | | 1.16 | 1.25 | 1009.57 | | 1020.63 | |  |
| Piecewise linear at a cut-off point of 28 °C | | | | | | | | | | | | | |  | |  | | |
| ≤ 28 °C of WBGT | | 295 | | 0.18 | | 0.12 | | 0.125 | | 1.20 | | 0.95 | 1.51 | 1009.04 | | 1023.79 | |  |
| > 28 °C of WBGT | | 295 | | 0.19 | | 0.02 | | <.001** | | 1.21 | | 1.15 | 1.26 |  | |  | |  |
| Piecewise linear at a cut-off point of 29 °C | | | | | | | | | | | | | |  | |  | | |
| ≤ 29 °C of WBGT | | 295 | | 0.22 | | 0.09 | | 0.011* | | 1.25 | | 1.05 | 1.47 | 1013.94 | | 1028.68 | |  |
| > 29 °C of WBGT | | 295 | | 0.18 | | 0.03 | | <.001** | | 1.20 | | 1.14 | 1.26 |  | |  | |  |
| Piecewise linear at a cut-off point of 30 °C | | | | | | | | | | | | | |  | |  | | |
| ≤ 30 °C of WBGT | | 295 | | 0.29 | | 0.07 | | <.001** | | 1.34 | | 1.16 | 1.53 | 1009.04 | | 1023.79 | |  |
| > 30 °C of WBGT | | 295 | | 0.15 | | 0.03 | | <.001** | | 1.16 | | 1.09 | 1.23 |  | |  | |  |
| Overall heat-related symptoms at 75^th^ cut-off point^b^ | | | | | | | | | | | | | |  | |  | | |
| Single linear | |  | |  | |  | |  | |  | |  |  |  | |  | |  |
| WBGT_eff_-FS-TWA (°C) | | 295 | | 0.38 | | 0.07 | | <.001** | | 1.47 | | 1.29 | 1.67 | 306.45 | | 313.82 | |  |
| Piecewise linear at a cut-off point of 28 °C | | | | | | | | | | | | | |  | |  | | |
| ≤ 28 °C of WBGT | | 295 | | 0.41 | | 0.52 | | 0.434 | | 1.51 | | 0.54 | 4.20 | 307.14 | | 318.20 | |  |
| > 28 °C of WBGT | | 295 | | 0.38 | | 0.07 | | <.001** | | 1.47 | | 1.27 | 1.69 |  | |  | |  |
| Piecewise linear at a cut-off point of 29 °C | | | | | | | | | | | | | |  | |  | | |
| ≤ 29 °C of WBGT | | 295 | | 0.52 | | 0.40 | | 0.194 | | 1.68 | | 0.77 | 3.65 | 309.40 | | 320.47 | |  |
| > 29 °C of WBGT | | 295 | | 0.37 | | 0.08 | | <.001** | | 1.45 | | 1.25 | 1.69 |  | |  | |  |
| Piecewise linear at a cut-off point of 30 °C | | | | | | | | | | | | | |  | |  | | |
| ≤ 30 °C of WBGT | | 295 | | 0.70 | | 0.32 | | 0.027* | | 2.01 | | 1.08 | 3.75 | 308.45 | | 319.51 | |  |
| > 30 °C of WBGT | | 295 | | 0.30 | | 0.10 | | 0.003* | | 1.35 | | 1.11 | 1.64 |  | |  | |  |
| Overall heat-related symptoms at median cut-off point^b^ | | | | | | | | | | | | | |  | |  | | |
| Single linear | |  | |  | |  | |  | |  | |  |  |  | |  | |  |
| WBGT_eff_-FS-TWA (°C) | | 295 | | 0.39 | | 0.05 | | <.001** | | 1.47 | | 1.33 | 1.64 | 335.39 | | 342.76 | |  |
| Piecewise linear at a cut-off point of 28 °C | | | | | | | | | | | | | |  | |  | | |
| ≤ 28 °C of WBGT | | 295 | | -0.10 | | 0.19 | | 0.585 | | 0.90 | | 0.63 | 1.30 | 331.48 | | 342.54 | |  |
| > 28 °C of WBGT | | 295 | | 0.48 | | 0.07 | | <.001** | | 1.61 | | 1.42 | 1.83 |  | |  | |  |
| Piecewise linear at a cut-off point of 29 °C | | | | | | | | | | | | | |  | |  | | |
| ≤ 29 °C of WBGT | | 295 | | 0.06 | | 0.14 | | 0.651 | | 1.07 | | 0.81 | 1.41 | 334.95 | | 346.02 | |  |
| > 29 °C of WBGT | | 295 | | 0.49 | | 0.07 | | <.001** | | 1.63 | | 1.41 | 1.87 |  | |  | |  |
| Piecewise linear at a cut-off point of 30 °C | | | | | | | | | | | | | |  | |  | | |
| ≤ 30 °C of WBGT | | 295 | | 0.22 | | 0.13 | | 0.09 | | 1.24 | | 0.97 | 1.59 | 335.41 | | 346.47 | |  |
| > 30 °C of WBGT | | 295 | | 0.49 | | 0.09 | | <.001** | | 1.64 | | 1.36 | 1.96 |  | |  | |  |
| Severe heat-related symptoms at 75^th^ cut-off point^b^ | | | | | | | | | | | | | | | | | |  |
| Single linear | | | | | | | | | | | | | | | | | |  |
| WBGT_eff_-FS-TWA (°C) | | 295 | | 0.40 | | 0.06 | | <.001 | | 1.49 | | 1.32 | 1.68 | 325.03 | | 332.40 | |  |
| Piecewise linear at a cut-off point of 28 °C | | | | | | | | | | | | | | | | | |  |
| ≤ 28 °C of WBGT | | 295 | | 0.17 | | 0.35 | | 0.626 | | 1.19 | | 0.59 | 2.38 | 326.68 | | 337.74 | |  |
| > 28 °C of WBGT | | 295 | | 0.42 | | 0.07 | | <.001** | | 1.52 | | 1.32 | 1.74 |  | |  | |  |
| Piecewise linear at a cut-off point of 29 °C | | | | | | | | | | | | | | | | | |  |
| ≤ 29 °C of WBGT | | 295 | | 0.31 | | 0.27 | | 0.261 | | 1.36 | | 0.80 | 2.33 | 328.37 | | 339.43 | |  |
| > 29 °C of WBGT | | 295 | | 0.41 | | 0.07 | | <.001** | | 1.51 | | 1.30 | 1.74 |  | |  | |  |
| Piecewise linear at a cut-off point of 30 °C | | | | | | | | | | | | | | | | | |  |
| ≤ 30 °C of WBGT | | 295 | | 0.60 | | 0.26 | | 0.021* | | 1.82 | | 1.10 | 3.03 | 326.27 | | 337.33 | |  |
| > 30 °C of WBGT | | 295 | | 0.33 | | 0.09 | | <.001** | | 1.40 | | 1.16 | 1.68 |  | |  | |  |
| Severe heat-related symptoms at median cut-off point^b^ | | | | | | | | | | | | | | | | | |  |
| Single linear | | | | | | | | | | | | | | | | | |  |
| WBGT_eff_-FS-TWA (°C) | | 295 | | 0.39 | | 0.05 | | <.001** | | 1.48 | | 1.33 | 1.64 | 344.64 | | 352.01 | |  |
| Piecewise linear at a cut-off point of 28 °C | | | | | | | | | | | | | | | | | |  |
| ≤ 28 °C of WBGT | | 295 | | 0.19 | | 0.26 | | 0.460 | | 1.21 | | 0.73 | 2.02 | 346.11 | | 357.18 | |  |
| > 28 °C of WBGT | | 295 | | 0.41 | | 0.06 | | <.001** | | 1.51 | | 1.34 | 1.71 |  | |  | |  |
| Piecewise linear at a cut-off point of 29 °C | | | | | | | | | | | | | | | | | |  |
| ≤ 29 °C of WBGT | | 295 | | 0.29 | | 0.19 | | 0.136 | | 1.33 | | 0.91 | 1.95 | 348.40 | | 359.46 | |  |
| > 29 °C of WBGT | | 295 | | 0.41 | | 0.07 | | <.001** | | 1.51 | | 1.32 | 1.73 |  | |  | |  |
| Piecewise linear at a cut-off point of 30 °C | | | | | | | | | | | | | | | | | |  |
| ≤ 30 °C of WBGT | | 295 | | 0.43 | | 0.17 | | 0.010* | | 1.54 | | 1.11 | 2.15 | 346.56 | | 357.62 | |  |
| > 30 °C of WBGT | | 295 | | 0.37 | | 0.09 | | <.001** | | 1.45 | | 1.22 | 1.73 |  | |  | |  |

WBGT_eff_-FS-TWA = full work shift time-weighted average effective Wet Bulb Globe Temperature; RR = Relative risk; OR = Odds ratios; S.E. = Standard error; ^a^Generalised linear negative binomial model; ^b^Generalised binary logistic model; *p-value < 0.05; **p-value < 0.001

Information described Table S9

Number of overall heat-related symptoms: The results suggested that there was evidence for potential non-linear relationship at the piecewise linear at a cut-off point of 28 and 29 °C (B = 0.02 and 0.15, respectively), with more pronounced effects above 28 and 29 °C (B = 0.13 and 0.13, respectively). However, the change in slopes at a cut-off point of 30 °C suggested a consistent linear pattern across the threshold because the change in the slopes was small (B = 0.10 for ≤ 30 °C and 0.12 for > 30 °C), with significant relationships both below and above.

Number of severe heat-related symptoms: The results suggested that there was minimal evidence of non-linearity at the piecewise linear at a cut-off point of 28 °C. For temperatures at or below 28°C, the effect was positive but not statistically significant (B = 0.18, p-value = 0.125, RR = 1.20), while for temperatures above 28°C, the effect was similarly positive and statistically significant (B = 0.19, p-value < 0.001, RR = 1.21). However, at the piecewise linear at a cut-off point of 29 and 30 °C, the results indicated significant non-linearity in the relationship between WBGT and number of severe heat-related symptoms because there were significant changes in the slopes (B) at both cut-off points. Interestingly, the impact of WBGT of the number of symptoms was greater at lower WBGT (B = 0.22 for ≤ 29 °C, and B = 0.29 for ≤ 30 °C), compared to higher WBGT (B = 0.18 and 0.15, respectively).

Overall heat-related symptoms at 75th cut-off point: The results indicated that there was little evidence of non-linearity at the piecewise linear at cut-off points of 28 and 29 °C as the effects were not significant at the lower temperatures of both cut-off points (p-value = 0.434 for ≤ 28 °C and 0.194 for ≤ 29 °C). However, the impact of WBGT on the number of symptoms was greater at lower WBGT (B = 0.42 for ≤ 28 °C, 0.52 for ≤ 29 °C, and 0.70 for ≤ 30 °C), compared to higher WBGT (B = 0.38, 0.37 and 0.30, respectively).

Overall heat-related symptoms at median cut-off point: The results indicated significant non-linearity in the relationship between the WBGT and the overall heat-related symptoms at mean cut-off point for the piecewise linear at all cut-off points, 28, 29, and 30°C. These results suggested that the WBGT had a small effect below the cut-off points (B = -0.10 for ≤ 28 °C, 0.06 for ≤ 29 °C, and 0.22 for ≤ 30 °C), but a strong positive effect above (B = 0.48, 0.49 and 0.49, respectively).

Severe heat-related symptoms at 75th cut-off point: The results suggested that there was significant non-linearity in the relationship between the WBGT and severe heat-related symptoms at piecewise linear models with cut-off points at 28°C and 29°C. The WBGT had less effect below the cut-off points for ≤ 28 °C (B = 0.17), but a strong positive effect above them (B = 0.42). However, at the piecewise linear at a cut-off point of 30 °C, the impact of WBGT on severe heat-related symptoms was greater at lower WBGT (B = 0.63), compared to higher WBGT (B = 0.33).

Severe heat-related symptoms at median cut-off point: The results suggested that there was evidence for potential non-linearity in the relationship between the WBGT and severe heat-related symptoms at the piecewise linear at a cut-off point of 28 and 29 °C (B = 0.19 and 0.29, respectively), with more pronounced effects above 28 and 29 °C (B = 0.41 and 0.41, respectively). However, at the piecewise linear at a cut-off point of 30 °C, the impact of WBGT on severe heat-related symptoms was greater at lower WBGT (B = 0.43), compared to higher WBGT (B = 0.37).

Table S10 The relationship between WBGT_eff_-FS-TWA (°C) categorical form and heat-related symptoms

| WBGT_eff_-FS-TWA (°C) | n | Unadjusted RR/OR (95% CI) |
| --- | --- | --- |
| Number of overall heat-related symptoms^a^ |  |  |
| > 32.00 | 157 | 2.16 (1.77 - 2.64) |
| 30.01 - 32.00 | 37 | 1.93 (1.52 - 2.46) |
| 28.01 - 30.00 | 62 | 1.09 (0.86 - 1.38) |
| ≤ 28.00 | 39 |  |
| Number of severe heat-related symptoms^a^ |  |  |
| > 32.00 | 157 |  |
| 30.01 - 32.00 | 37 | 4.63 (2.96 - 7.23) |
| 28.01 - 30.00 | 62 | 3.98 (2.42 - 6.53) |
| ≤ 28.00 | 39 | 1.66 (1.00 - 2.76) |
| Overall heat-related symptoms at 75^th^ cut-off point^b^ |  |  |
| > 32.00 | 157 | 26.85 (3.59 - 200.54) |
| 30.01 - 32.00 | 37 | 18.24 (2.23 - 149.18) |
| 28.01 - 30.00 | 62 | 3.33 (0.38 - 29.66) |
| ≤ 28.00 | 39 |  |
| Overall heat-related symptoms at median cut-off point^b^ |  |  |
| > 32.00 | 157 | 12.06 (5.23 - 27.82) |
| 30.01 - 32.00 | 37 | 6.94 (2.52 - 19.15) |
| 28.01 - 30.00 | 62 | 1.36 (0.54 - 3.44) |
| ≤ 28.00 | 39 |  |
| Severe heat-related symptoms at 75^th^ cut-off point^b^ |  |  |
| > 32.00 | 157 | 18.27 (4.26 - 78.41) |
| 30.01 - 32.00 | 37 | 12.61 (2.63 - 60.44) |
| 28.01 - 30.00 | 62 | 1.98 (0.38 - 10.36) |
| ≤ 28.00 | 39 |  |
| Severe heat-related symptoms at median cut-off point^b^ |  |  |
| > 32.00 | 157 | 14.99 (5.53 - 40.64) |
| 30.01 - 32.00 | 37 | 8.93 (2.85 - 27.96) |
| 28.01 - 30.00 | 62 | 2.17 (0.72 - 6.55) |
| ≤ 28.00 | 39 |  |

^a^Generalised linear negative binomial model with unadjusted RR (Relative risk); ^b^Generalised binary logistic model with unadjusted OR (Odds ratios); WBGT_eff_-FS-TWA = full work shift time-weighted average effective Wet Bulb Globe Temperature
